# Expression of Concern: The silencing of long non-coding RNA ANRIL suppresses invasion, and promotes apoptosis of retinoblastoma cells through ATM-E2F1 signaling pathway

**DOI:** 10.1042/BSR-20180558_EOC

**Published:** 2021-03-26

**Keywords:** ATM-E2F1 signaling pathway, Cell apoptosis, Cell proliferation, Long-non coding RNA ANRIL, Negative feedback regulation, Retinoblastoma

The Editorial Office has been made aware of potential issues surrounding the scientific validity of this paper, hence has issued an expression of concern to notify readers whilst the Editorial Office investigates.

